# Development of citrinin-loaded liposomes and in vitro toxicogenetic evaluation on breast cells

**DOI:** 10.1007/s00210-026-05695-x

**Published:** 2026-07-21

**Authors:** Ag-Anne Pereira Melo de Menezes, Maria Luísa Lima Barreto do Nascimento, Antonielly Campinho dos Reis, Michely Laiany Vieira Moura, Taline Alves Nobre, Athanara Alves de Sousa, Jorddam Almondes Martins, Sérgio Eduardo Matos Cazarotti Francisco, João Pedro Alves Damaceno do Lago, Igor Gabriel Barbosa de Sousa, Helber Alves Negreiros, Marjorie Pereira Gualter, Débora Cavalcante Braz, Juan Carlos Ramos Gonçalves, Marianna Vieira Sobral, Karinne Kelly Gadelha Marques, Isione Oliveira Castro, Thais Cruz Ramalho, Paulo Michel Pinheiro Ferreira, Dalton Dittz, Bruna da Silva Souza, Felipe Cavalcanti Carneiro da Silva, Hercilia Maria Lins Rolim, João Marcelo de Castro e Sousa

**Affiliations:** 1 Laboratory of Research in Toxicological Genetics (LAPGENIC), Department of Biochemistry and Pharmacology, Federal University of Piauí, Teresina, Piauí 64.049-550 Brazil; 2 Department of Pharmacy, Federal University of Piauí, Teresina, Piauí 64.049-550 Brazil; 3 Laboratory of Oncopharmacology (ONCOFAR), Federal University of Paraíba, João Pessoa, Paraíba 64.202-020 Brazil; 4 Laboratory of Experimental Oncology (LOE), Department of Physiology and Pharmacology, Federal University of Ceará, Fortaleza, Ceará 60.430-275 Brazil; 5 Laboratory of Antineoplasic Pharmacology (LAFAN), Department of Biochemistry and Pharmacology, Federal University of Piauí, Teresina, Piauí 64.049-550 Brazil; 6 Laboratory of Biochemistry for Dairy Plants (LABPL), Federal University of Delta of Parnaíba, Parnaíba, Piauí 64.202-020 Brazil; 7 Laboratory of Pharmaceutical Nanosystems (NANOSFAR), Postgraduate Program in Pharmaceutical Sciences, Teresina, Piaui Brazil

**Keywords:** Nanotechnology, Pharmaceutical formulations, Neoplasms, Mycotoxin, Toxicogenetic effects

## Abstract

This study aimed to synthesize a liposomal nanoformulation containing citrinin (LP-CIT) to improve its availability and to evaluate its toxicogenomic profile, focusing on cytotoxic and genotoxic effects in breast cancer (MDA-MB-231) and non-tumorigenic mammary epithelial (MCF-10A) cell lines. The formulation was characterized by macroscopic appearance, pH, particle size, polydispersity index (PDI), zeta potential, encapsulation efficiency, and estability of nanoparticles. Citrinin content, encapsulation efficiency, and liposome stability were evaluated. Cytotoxicity was assessed using MTT, trypan blue exclusion, and flow cytometry. Cell death mechanisms were investigated by mitochondrial membrane potential analysis and expression of apoptotic markers (caspase-9 and BAX). Genotoxicity was evaluated using the comet assay. The liposomal nanosystem consisted of small unilamellar vesicles with a mean diameter of 143.3 nm, a PDI of 0.3, and a positive surface charge (+ 28.5 mV). The average was 63.69 ± 0.18% of citrinin encapsulated in liposomes with stability more than 21 days. Both free citrinin (CIT) and LP-CIT showed cytotoxic effects in MDA-MB-231 and MCF-10A cells, with lower LC₅₀ values observed for LP-CIT. Although LP-CIT induced more pronounced cytotoxic and genotoxic effects in the cancer cell line, toxic effects were also observed in non-tumorigenic cells, indicating limited selectivity. Cell death was associated with mitochondrial membrane depolarization and increased caspase-9 and BAX expression. Both formulations induced DNA damage as detected by the comet assay. Liposomal encapsulation enhanced citrinin biological activity but did not indicate tumor selectivity. Thus, this study represents a toxicogenomic assessment of citrinin and its liposomal formulation for potential therapeutic applicability.

**Supplementary Information:**

The online version contains supplementary material available at https://doi.org/10.1007/s00210-026-05695-x.

## Introduction

The challenge with current cancer therapeutic strategies is the creation of drugs for a specific target, which has the ability to improve selectivity and reduce side effects in healthy tissues (Lee et al. 2017). Traditional cancer therapies have low solubility, high metabolism, and hydrophobia, which enhance systemic toxicity and limit availability. To address these issues, nanoformulations are being developed to improve targeted distribution and effectiveness while minimizing adverse effects (Chiang et al. 2021; Hwang et al. 2021).

Liposomes are the most often employed nanocarriers in targeted drug delivery systems (Large et al. 2021). These nanocarriers offer benefits for overcoming cellular absorption barriers and increasing drug biodistribution (Monteiro et al. 2018). Liposomes with diameters ranging from 100 to 200 nm can penetrate tumors and be retained for an extended period of time due to improved permeability and retention (Wen et al. 2018).

Liposomes represent one of the most effective drug carriers and delivery systems utilized in cancer therapy, encompassing formulations such as doxorubicin liposomes (Doxil), daunorubicin liposomes (DaunoXome), cytarabine liposomes (Depocyt), vincristine liposomes (Marqibo), and non-pegylated doxorubicin liposomes (Myocet) (Mu et al. 2017; Knudsen et al. 2015). The use of liposomes may enhance the pharmacokinetic and pharmacodynamic properties of innovative anticancer compounds, particularly those derived from natural products.

Over the last four decades, natural products, both in their native and modified forms, have played an important role in cancer therapy (Colone et al. 2020). Among these compounds, citrinin (CIT), a mycotoxin isolated from Penicillium citrinum, has demonstrated cytotoxic and genotoxic activity in tumor cells in vitro and in cancer models in vivo. Previous studies have reported that CIT exerts antitumor effects through mechanisms involving the modulation of oxidative stress, the induction of apoptosis, and cell cycle arrest. However, its therapeutic applicability remains limited due to dose-dependent systemic toxicity and the absence of targeted administration approaches (Oliveira et al. 2020a; Salah et al. 2019; Babu et al. 2017; Oliveira et al. 2021; Singh et al. 2016).

However, CIT has limitations related to hepatotoxicity and nephrotoxicity when administered at high concentrations (Zhang et al. 2020). CIT induces nephrotoxicity by mechanisms associated with cell cycle arrest and microtubule disruption, as well as alterations in p53 and p21 expression that interfere with cell division (Chang et al. 2009). Despite these limitations, CIT has demonstrated significant cytotoxic activity in tumor models and is therefore investigated in this study as a bioactive lead compound rather than as a ready-to-use systemic therapeutic agent. Previous in vivo studies evaluating liposomal citrinin demonstrated enhanced antitumor activity accompanied by indications of reduced systemic toxicity compared with the free compound, including improved tolerability and a more favorable toxicological profile, supporting the rationale for nanoencapsulation as a strategy to mitigate hepatotoxic effects (Moura et al. 2024); however, such parameters were not directly evaluated in the present in vitro study. In this context, the incorporation of citrinin into liposomal nanosystems was explored as a strategy to modulate its biodistribution, reduce free systemic exposure, and potentially mitigate toxicity while enhancing tumor-associated effects through targeted delivery. Thus, the present study aimed to develop liposomal formulations containing CIT and evaluate their cytotoxic and genotoxic effects in mammary tumor cells as a proof-of-concept toxicogenomic assessment.

## Methods

### Preparation of liposomal formulation containing citrinin

Liposomes containing citrinin were prepared using the lipid film hydration method with adaptations (Anchiêta-Junior 2014); Moreno et al. 2014). Citrinin (CIT) as well as the reagents for liposome preparation were > 95% pure (Sigma-Aldrich, USA). Initially, conventional liposomes containing citrinin (LP-CIT), positively charged with soybean phosphatidylcholine lipids (Lipoid S 100), cholesterol, and octadecylamine (octadecylamine-cationic lipid) (ratio of 70:20:10), and a citrinin concentration of 0.1 mg/mL were produced to obtain a liposomal formulation with a lipid concentration of 42 mM. The constituents were solubilized in a mixture of chloroform and methanol (3:1 v/v) under magnetic stirring. The solvents were removed by vacuum evaporation using a rotaevaporator for 40 min (37 ± 1 °C, 80 rpm). The lipid film was hydrated with 10 mL of phosphate buffer solution at pH 7.4 to form large multilamellar vesicles. For storage, lyophilization was the method of choice, where 1 g of trehalose with phosphate buffer was added to the formulation. The lipossomal suspension was then subjected to sonication by ultrasonic probe Ultronique Ultrasonic Probe (Eco-sonics, model QR500) with frequenc of 40 kHz and power of 200 Watts during 5 min to obtain small unilamellar liposomes containing citrinin and previously called LP-CIT.

### Physical and chemical characterization

The parameters evaluated were macroscopic appearance, pH, particle size (PS), polydispersity index (PDI), and zeta potential (ZP). The liposome pH was measured using a digital pH meter (Bioblock Scientific 99.622, Prolabo, Paris, France) at room temperature. The liposomes PS and PDI were determined using photon correlation spectroscopy (Beckman Coulter Delsa ™ Nano S particle analyzer). For PDI and PS analysis, 300 μL of liposomal suspension was diluted in 1 mL of ultrapure water. The zeta potential of the liposomes, which corresponds to the surface charge of the vesicles, was determined by diluting 20 μL of the liposomal suspension in 1 mL of ultrapure water and reading the resulting solution on a Zetatrac NC-148 apparatus (Microtrac). The test was performed in triplicate.

### Determination of citrinin content in liposomes

The quantification of citrinin incorporated in liposomes was carried out by Cary 60 UV–Vis spectrophotometer (Agilent, USA). Quantification was performed using the external standard method, and a calibration curve was constructed with citrinin concentrations of 0.38, 0.75, 1.25, 2.5, 5.0, and 20.0 μg/mL, in triplicate, and linear regression. The calibration curve for determining encapsulation efficiency was established in metanol. Data were collected by monitoring precursor and productions simultaneously. A Waters Acquity XBridge TM BEH C18 2.5 µm, 2.1 × 100 mm XP column was used with a mobile phase consisting of A (0.1% formic acid, v/v) and B (methanol) in a gradient system: 0–4 min, 5–100% A, and 0.5 mL/min flow rate. The column was equilibrated for 2 min before each injection. Column temperature and injection volume were set to 25 °C and 20 μL, respectively. All analyses were performed using MassLynx software.

Quantification was performed by the external standard method, and a calibration curve was constructed with citrinin concentrations of 0.38, 0.75, 1.25, 2.5, 5.0, and 20.0 ppm, in triplicate, and linear regression. A calibration curve for free citrinin was prepared with concentrations ranging from 0.3125 to 20 µg/mL in mass grade methanol.

### Encapsulation efficiency

The citrinin encapsulation efficiency was measured using a ultrafiltration/ultracentrifugation process with Ultrafree® units (Millipore, USA). A 1 mL aliquot of liposomal material was transferred to Millipore® filter units and ultracentrifuged for 1 h at 10,000 rpm. After centrifugation, the filtrate was dosed in UV–Vis spectrophotometer. The amount of encapsulated citrinin was calculated as the difference between the total amount measured in the formulation and that obtained in the filtrate (Lira et al. 2009). To assess efficiency, the analytical curve was ran in methanol at concentrations ranging from 0.3125 to 20 µg/mL. The analytical curve was evaluated using the least squares regression method to verify its linearity and correlation coefficient. The linear line equation was used to quantify the drug in the sample supernatant containing the encapsulated medication. The test was carried out in triplicate.

### Preliminary evaluation of stability

For formulations monitoring, preliminary stability was assessed after preparing the formulations. In addition, formulation pH and homogeneity were monitored over the days. The parameters evaluated were macroscopic appearance, pH variation, particle size, and polydispersity index (PDI) for a period of 21 days (Anchiêta-Junior 2014); Moreno et al. 2014; Lira et al. 2009). After this preliminary analysis, the liposomes were stored in lyophilized form. For lyophilization, 1 g of trehalose solubilized with phosphate buffer was added to the formulation. The test was performed in triplicate.

### Cell culture and chemicals

The cell line MDA-MB-231 (human breast adenocarcinoma line) was provided by the Federal University of Minas Gerais (UFMG). Dulbecco’s Modified Eagle Medium (DMEM) (Gibco®, Invitrogen, USA) was used for MCF-10A cells, and RPMI-1640 medium (GIBCO®, Invitrogen, USA) was used for MDA-MB-231. All cell lines were supplemented with 10% (v/v) fetal bovine serum and 1% (v/v) penicillin/streptomycin. The cell lines were grown in bottles and maintained in an incubator with a 5% CO_2_ atmosphere at 37 °C. At 90% confluence, cells were washed with PBS/EDTA pH 7.4 and trypsinized (0.5 mL). For the assays, the cell lines were treated with free citrinin and liposome-encapsulated citrinin, as well as empty liposomes and negative (medium only) and positive (DOX at 2 µg/mL) controls. The exposure time was 72 h, and the concentrations varied according to the assay.

### Analysis of cell viability by MTT assay

The MTT assay {[3-(4,5-dimethylthiazol-2yl)−2,5-diphenyl tetrazolium bromide]} was performed according to Mosmann (1983). The cells were distributed in 96-well plates and treated with the controls, free CIT, and the liposomal formulations, with a concentration range between 30 and 0.11 µg/mL. They were then incubated for 72 h to determine the LC_50_. After the incubation period, 20 µL of MTT (5 mg/mL solution) was added to the cultures and incubated for 4 h in a CO_2_ incubator. After removal of the supernatant, the formazan crystals were dissolved in 100 μL of DMSO for an additional 30 min, and the absorbances were measured in a microplate reader (Microplate Reader EZ Read 400) at a wavelength of 570 nm. For this bioassay, the concentration capable of inducing 50% cell cytotoxicity (CL_50_) in each cell line was calculated and estimated from the results of the cell viability assay, considering the viability of the control group as 100%. All MTT assays were performed in three independent biological experiments (*n* = 3), each conducted in technical triplicate for each concentration and condition.

Furthermore, the tumor selectivity index (TSI) was also calculated, following the formula TSI = IC50 (normal cell line)/IC50 (tumor cell line). A TSI greater than 1 suggests that citrinin and/or its nanoformulations are selective for the evaluated tumor cell line.

### Trypan Blue cell viability

Cell viability was also determined using a trypan blue exclusion assay adapted from Strober (2015). MCF-10A and MDA-MB-231 cell lines were seeded in 24-well plates at a density of 5 × 10^5^ cells. After 24 h, the culture media was replaced, and the cells were treated in triplicate with free citrinin (CIT: 0.1, 1 and 5 µg/mL), liposomal formulations (LP-CIT: 0.1, 1 and 5 µg/mL), empty liposomes (EL: 5, 10, and 20 µg/mL), and negative (medium) and positive controls (DOX, 2 µg/mL). After 72 h exposure, 10 μL of the cell suspension was removed from the cultures, and 10 μL of trypan blue was added to each treatment group. A × 400 optical microscope was employed in conjunction with a Neubauer chamber. The proportion of viable cells was calculated with the following formula:

$$Viable\;cells\;\left(\%\right)=\frac{Total\;numer\;of\;viable\;per\;milliliter\;of\;aliquot\;\times\;100}{Total\;numer\;os\;cells\;per\;milliliter\;of\;aliquot\;\left(viable\;\vee\;not\right)}$$

### Assessment of cell death by flow cytometry

To determine the percentage of viable, apoptotic or necrotic cells, MDA-MB-231 tumor cells and non-tumor MCF-10A cells (72 h exposure time) were added to one unlabeled tube, one positive control tube, and one tube for each treatment used: free citrinin (CIT: 0.1, 1 and 5 µg/mL), liposomal formulations (LP-CIT: 0.1, 1 and 5 µg/mL), empty liposomes (EL: 20 µg/mL), and negative and positive controls (DOX, 2 µg/mL). The tubes were centrifuged to remove the medium, and 100 µL of 0.9% saline was added and homogenized in a vortex. Then, 2 µL of Annexin V PE (AV) and 2 µL of 7-AAD from the PE Annexin V Apoptosis Detection Kit I BD Pharmigen® were added to the tubes and incubated in a dark environment for 30 min. After this period, 300 µL of saline was added, and 30,000 events for each treatment (triplicates of 10,000 events) were acquired and recorded on a FACSCanto II® flow cytometer. Analysis of viable and non-viable cells is done by fluorescence intensity so that live cells are represented by the negative peak, and the positive fluorescence intensity peak represents dead cells. Events were classified as follows: viable events (Annexin -/7-AAD), events in early apoptosis (Annexin +/7-AAD-), events in late apoptosis (Annexin +/7-AAD +), and events in necrosis (Annexin-/7-AAD +) (Marcondes et al. 2019). Flow cytometry assays were performed in at least three independent biological replicates. Data acquisition and analysis were conducted by the same researcher who performed the experiments. Although the researcher was aware of the treatment groups, all analyses followed standardized protocols to ensure consistency and minimize bias.

#### Assessment of mitochondrial membrane potential (JC-1)

Staining of cells in suspension for the assessment of mitochondrial membrane potential was performed by incubating 1 × 106 cells/mL in 0.5 mL of JC-1 reagent in an incubator with 5% CO_2_ and + 37 °C for 15 min. Centrifugation was then performed for 5 min at 400 × *g* and the supernatant was removed. The cell pellet was resuspended in 2 mL of buffer followed by centrifugation (Reers et al. 1995; Ortiz-Lazareno et al. 2014). The supernatant was removed. The cell pellet was resuspended in 0.3 mL of assay buffer. It was immediately observed with a fluorescence microscope using a dual-bandpass filter designed to simultaneously detect fluorescein and rhodamine or fluorescein. The test performed in triplicate. Fluorescence intensity was quantified by measuring red (J-aggregates) and green (monomers) signals using image analysis software (e.g., ImageJ), and mitochondrial membrane potential was expressed as the ratio of red to green fluorescence. Data were obtained from three independent experiments and expressed as mean ± SD.

### Comet test alkaline version

The alkaline version of the comet assay was performed as described by Speit and Rothfuss (2012). After 72 h, 10 µL aliquots of MDA-MB-231 and MCF-10A cell suspension were mixed with a thin layer of 0.75% low melting point agarose (90 µL) and placed on slides pre-coated with 1.5% normal agarose. Doxorubicin (2 µg/mL) was included as a positive control to validate the sensitivity and performance of the comet assay. The slides were then immersed in lysis solution (2.5 M NaCl, 100 mM EDTA, and 10 mM Tris, pH 10, with the addition of 1% Triton X-100 and 10% DMSO at the time of use) for up to 72 h at 4 °C. After this period, the slides were incubated in alkaline buffer (300 mM NaOH and 1 mM EDTA, pH > 13) for 20 min and then exposed to an electric current of 300 mA and 25 V (0.90 V/cm) for 15 min in an electrophoresis chamber. At the end, the slides were neutralized with Tris buffer (0.4 M and pH 7.5) and stained with silver solution. The slides were analyzed in an optical microscope (× 400 magnification), and the results were expressed as damage index (DI) and frequency of damage (FD) of 100 cells in triplicate per treatment. The DI was calculated using the formula DI = Σ (number of cells in each damage class × damage class), which ranged from 0 to 400, and FD by the following formula FD = 100 − number of cells in class 0. Comet assay analysis was performed by visual scoring, in which nucleoids were classified into five damage classes (0–4) according to tail length and intensity. For each experimental condition, at least 100 nucleoids were evaluated per experiment. All slides were analyzed by a single trained observer, following standardized criteria. Experiments were performed in at least three independent biological replicates. While the observer was aware of the treatment groups, scoring followed strict, predefined criteria to minimize bias.

### Western blotting

Tumor and normal cells were plated at a confluence of 3.5 × 10^5^/well in the 6-well multiplate and exposed to a 1.5 µg/mL concentration for time intervals of 2, 4, 8, and 12 h. Protein extraction was then performed using the lysis solution (mixture of 0.05 M Tris–HCl, 0.01 M EGTA, 0.001 M EDTA, 0.016 M Triton X-100, 0.001 M sodium orthovanadate, 0.05 M sodium fluoride, 0.01 M sodium pyrophosphate, and 0.25 M sucralose, diluted in distilled water and pH adjusted to 7.5). All reagents were purchased from Sigma-Aldrich (St. Louis, MO, USA) and added as per tablet use of a protease inhibitor cocktail (Roche Diagnostics; Indianapolis, IN, USA). Protein quantification was performed by Bradford methodology (Protein assay dye concentrate, Bio-Rad Laboratories; Hercules, CA, USA).

Protein lysates were subjected to SDS-PAGE, electrophoresed on a 12% polyacrylamide gel, electrotransferred to a polyvinylidene difluoride membrane, labeled with specific antibodies: HSP90 (constitutive chaperone), caspase-9, and BAX, all purchased from Cell Signaling®, and revealed by radiographic film. The test was performed in triplicate.

### Statistical analysis

All analyses were performed using GraphPad Prism software (version 7.0). The analyses were performed using one-way or two-way analysis of variance (ANOVA) followed by Tukey, considering < 0.05*, < 0.01**, and < 0.001***, with a 95%, 99%, and 99.9% confidence level, respectively. Results were expressed as mean ± standard deviation (SD).

## Results

### Particle size (PS) and zeta potential (ZP) of LP-CIT

**Fig. 1:**
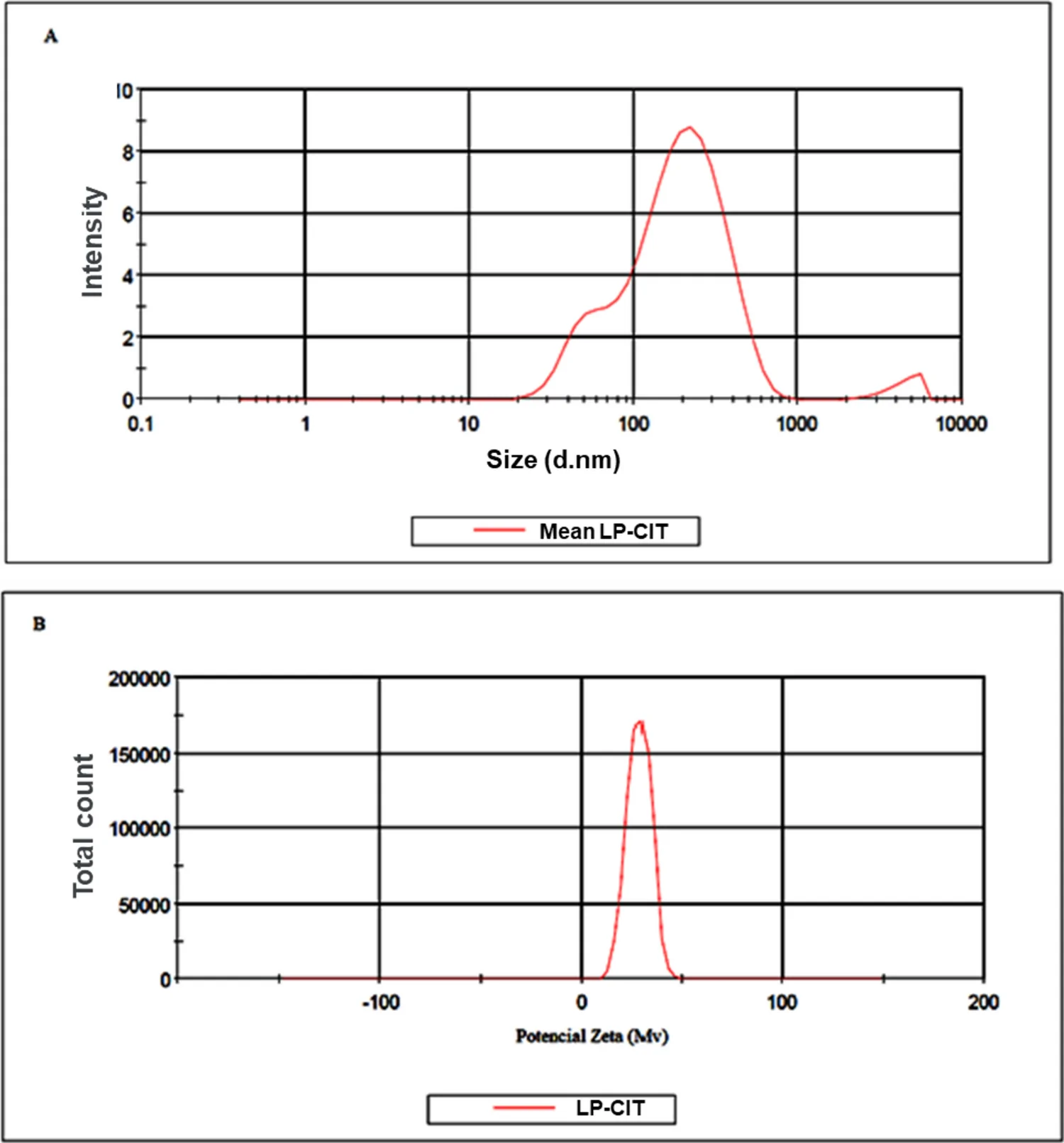
Physicochemical parameters of liposome containing citrinin. **A** Particle size distribution of LP-CIT. **B** Zeta potential of liposomes containing citrinin (LP-CIT)

Liposomes containing citrinin (LP-CIT) showed a polydispersity index (PDI) of 0.3, indicating a narrow particle size distribution, and the hydrodynamic diameter, measured by dynamic laser light scattering (DLS), was 143.3 nm (A). The zeta potential of LP-CIT was evaluated at + 28.5 mV (Fig. 1B). The results indicate that the liposomes are small unilamellar vesicles (SUV), with PS, PDI, and ZP compatible with applications in in vitro and in vivo tests.

The morphological behavior of liposomal citrinin compared to empty liposomes agrees with the result of the stability test for particle size and polydispersity index. After 21 days of storage, only slight variations in particle size and PDI were observed (Table 1), with no evidence of macroscopic aggregation or marked physicochemical instability. The formulations were stored in PBS (pH 7.4) at 4 °C during the stability evaluation period. To maintain the physicochemical characteristics, the nanosystems were stored in lyophilized form and resuspended with phosphate buffer at pH 7.4. Comparing the values, it can be observed that the largest diameter was obtained for the liposomes containing citrinin. This is indicative of citrinin encapsulation inside the vesicles, confirmed by the encapsulation efficiency. The values found for PDI indicate that the formulation containing citrinin presents particles with greater homogeneity compared to empty liposomes, which contributes to the stability and efficiency of these carriers for in vitro and in vivo tests (Table 1). Although morphological characterization by transmission electron microscopy (TEM) or scanning electron microscopy (SEM) was not performed in this study, future investigations will include these analyses to confirm vesicle lamellarity and structural integrity.

**Table 1:** Characteristics of liposomal formulations containing citrinin (LP-CIT)

	**EL (Day 01)**	**EL (Day 21)**	**LP-CIT (Day 01)**	**LP-CIT (Day 21)**
PS	123.3 ± 3.20 nm	128.7 ± 2.84 nm	143.3 ± 6.38 nm	155.4 ± 8.37 nm
PDI	0.304 ± 0.018	0.361 ± 0.021	0.390 ± 0.034	0.380 ± 0.052
ZP	+ 32.8 ± 1.39 mV	+ 35.6 ± 1.43 mV	+ 28.5 ± 5.79 Mv	+ 31.6 ± 4.62 Mv
pH	7.4	7.4	7.4	7.4

*TP*, particle size; *PDI*, polydispersity index; *PZ*, zeta potential; *pH*, hydrogen potential; *LP*, empty liposomes; *LP-CIT*, liposomes containing citrinin. Mean (*n* = 3) ± SE.

**Fig. 2:**
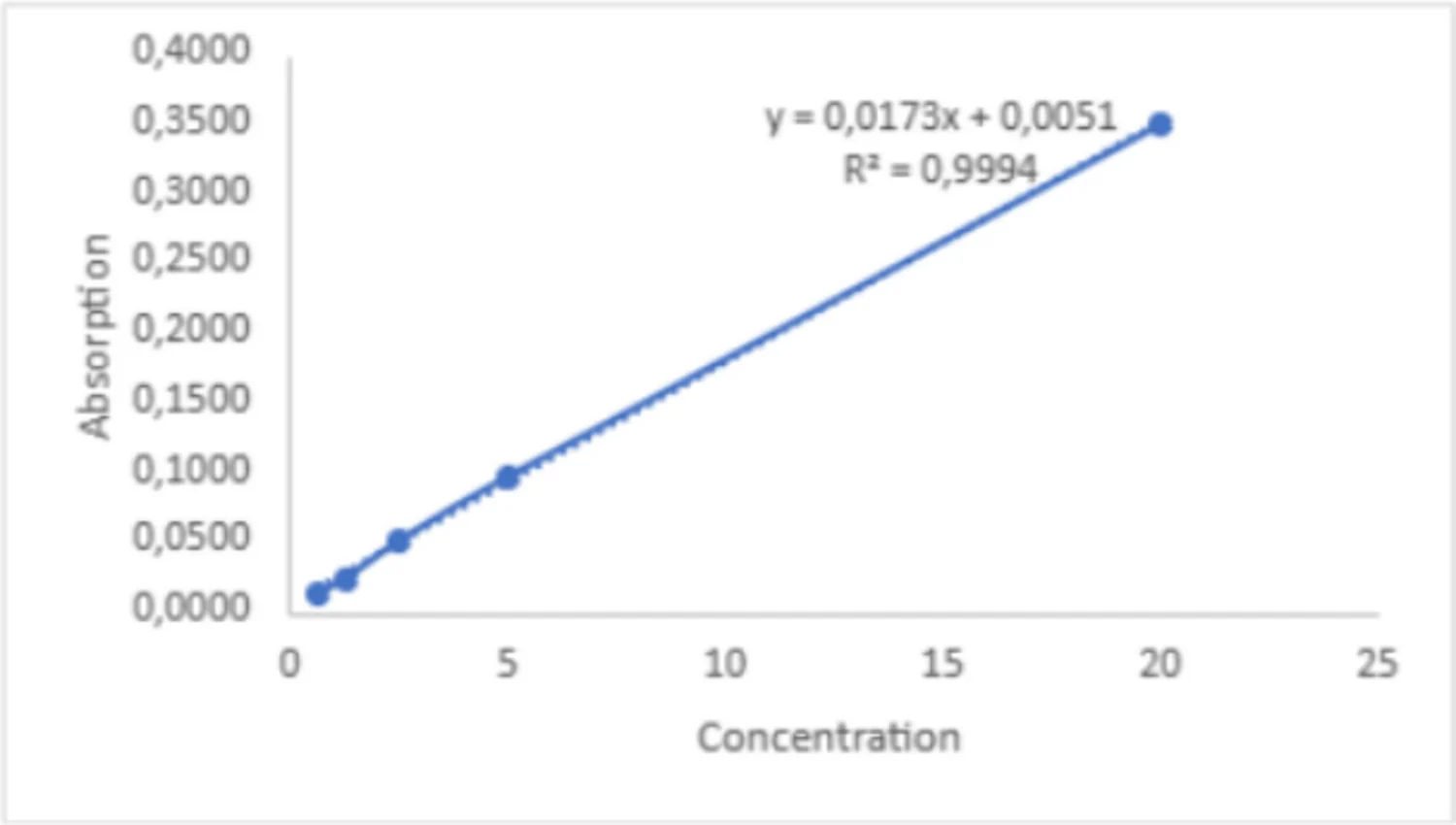
Citrinin analytical curve for the concentration range 0.3125 to 20 µg/mL

To determine the efficiency, the analytical curve was performed in concentrations ranging from 0.3125 to 20 µg/mL in methanol and presented a correlation coefficient of 0.9996 (Fig. 2).

Analytical calibration curve for citrinin obtained by the external standard method in the concentration range of 0.38–20.0 ppm. Each point represents the mean of triplicate measurements, and linear regression was used for quantification.

The citrinin-encapsulated liposomes were measured using the calibration curve seen in Fig. 2.

To find the citrinin concentration in the filtrate, the absorbance peaks were correlated with the equation of the calibration curve. As an aliquot of 300 µL (from the total 1 mL formulation) was used for the test, a correlation was established to determine the concentration in 1 mL. In 300 µL, the measured drug mass detected was 2.73 µg. The detected volume was then related to the total formulation volume (1 mL) to calculate the drug encapsulation efficiency in liposomes. The CIT encapsulation efficiency was 63.69 ± 0.18% in conventional positively charged liposomes, indicating satisfactory encapsulation for this nanosystem. The drug loading (DL) was calculated as 4.54%, representing the proportion of citrinin effectively incorporated relative to the total lipid content of the formulation.

#### CIT and LP-CIT cytotoxicity in tumor and non-tumor breast cells

MTT analyses revealed cytotoxic effects for free citrinin (CIT) in all two cell lines evaluated, presenting LC_50_ values of 10.56 and 9.18 µg/mL for MDA-MB-231 and MCF-10A, respectively. LP-CIT showed lower LC_50_ values (0.77 and 0.20 µg/mL) when compared to CIT. For the same bioassay, empty liposome (EL) also showed cytotoxicity with the following CI_50_ values: 10.31 and 9.18 µg/mL, respectively. Regarding doxorubicin cytotoxicity, MDA-MB-231 and MCF-10A had similar values (Table 2). Based on the TSI calculation, we obtained the following treatment indices: CIT 0.86; LP-CIT 0.25; EL 0.89; and DOX 1.00. Therefore, no selectivity was observed for the treatments.

**Table 2:** Cytotoxicity of free citrinin (CIT), empty liposomes (EL), and liposome containing citrinin (LP-CIT) on non-tumor and tumor cell lines

Cell lines			LC_50_ (µg/mL)^a^	
	CIT	LP-CIT	EL	DOX
MDA-MB-231	10.56	0.77	10.31	2.76
	(8.42–13.23)	(0.58–1.02)	(8.43**–**12.59)	(2.22–3.43)
MCF-10A	9.18	0.20	9.18	2.76
	(7.44–11.09)	(0.05–0.71)	(7.44–11.09)	(2.51–2.93)

^a^Values are expressed as LC_50_ and 95% (*p* < 0.05) confidence interval in parentheses. *EL*, empty liposome; *CIT*, free citrinin; *LP-CIT*, liposomal citrinin

The viability assay utilizing trypan blue on MCF-10A and MDA-MB-231 cells demonstrated that after 72 h of exposure to CIT and LP-CIT, both formulations presented cytotoxicity at all three tested concentrations, significantly reducing the viability of the non-tumor line MCF-10A (CIT 70.82, 72.11, and 21.74%; LP-CIT 90.63, 82.98, and 35.40%) and the MDA-MB-231 tumor cell line (CIT 83, 56.1, and 12.8%; LP-CIT 70.9, 35.6, and 3.3%). It was observed in MDA-MB-231 tumor cells that all LP-CIT concentrations had significantly increased cytotoxicity when compared to the same CIT concentrations. Moreover, it was observed in the MCF-10A non-tumor cell line that CIT had significantly increased cytotoxicity when compared to the same LP-CIT concentrations. The empty liposome (EL) only at the concentration of 20 µg/mL showed cytotoxic effects when compared to the negative control. All CIT, LP-CIT, and EL concentrations showed higher viability compared to DOX, except for LP-CIT (5 µg/mL) in MDA-MB-231 (Table 3).

**Table 3:** Cell viability by trypan blue bioassay in mammary tumor cell lines treated with free citrinin (CIT 0.1, 1, and 5 µg/mL), liposomal formulations (LP-CIT 0.1, 1, and 5 µg/mL), empty liposome (EL 5, 10, and 20 µg/mL), and negative and positive control (DOX, 2 µg/mL)

Treatment	Concentration (µg/mL)	MCF-10A	MDA-MB-231
NC	-	92.45 ± 1.2	94.25 ± 1.2
DOX	2	8.24 ± 0.8^a^	3.58 ± 0.3^a^
EL	5	90.45 ± 0.5^b^	91.69 ± 1.1^b^
	10	90.66 ± 0.8^b^	90.78 ± 2.4^b^
	20	88.88 ± 1.4^b^	47.12 ± 0.4^ab^
CIT	0.1	70.82 ± 5.0^ab^	83.08 ± 2.4^ab^
	1.0	72.11 ± 1.2^ab^	56.12 ± 4.1^ab^
	5.0	21.74 ± 5.72^ab^	12.83 ± 0.5^ab^
LP-CIT	0.1	90.63 ± 1.2^bc^	70.98 ± 1.7^abc^
	1.0	82.98 ± 1.6^abc^	35.62 ± 1.1^abc^
	5.0	35.40 ± 2.4^abc^	3.37 ± 0.7^ac^

Values were expressed by mean ± standard deviation using one-way ANOVA with Tukey’s post hoc test, with *p*-values < 0.05 statistically significant.

^a^Different when compared to NC.

^b^Different when compared DOX.

^c^LP-CIT different when compared to CIT at the same concentration.

*DI* damage index (0–400). *FD* damage frequency (%). *NC* untreated cells. *DOX* cells treated with doxorubicin (2 µg/mL). *CIT* free citrinin. *LP-CIT* liposome containing citrinin. *EL* empty liposome.

### CIT and LP-CIT induce cell death in mammary tumor and non-tumor mammary cells

**Fig. 3:**
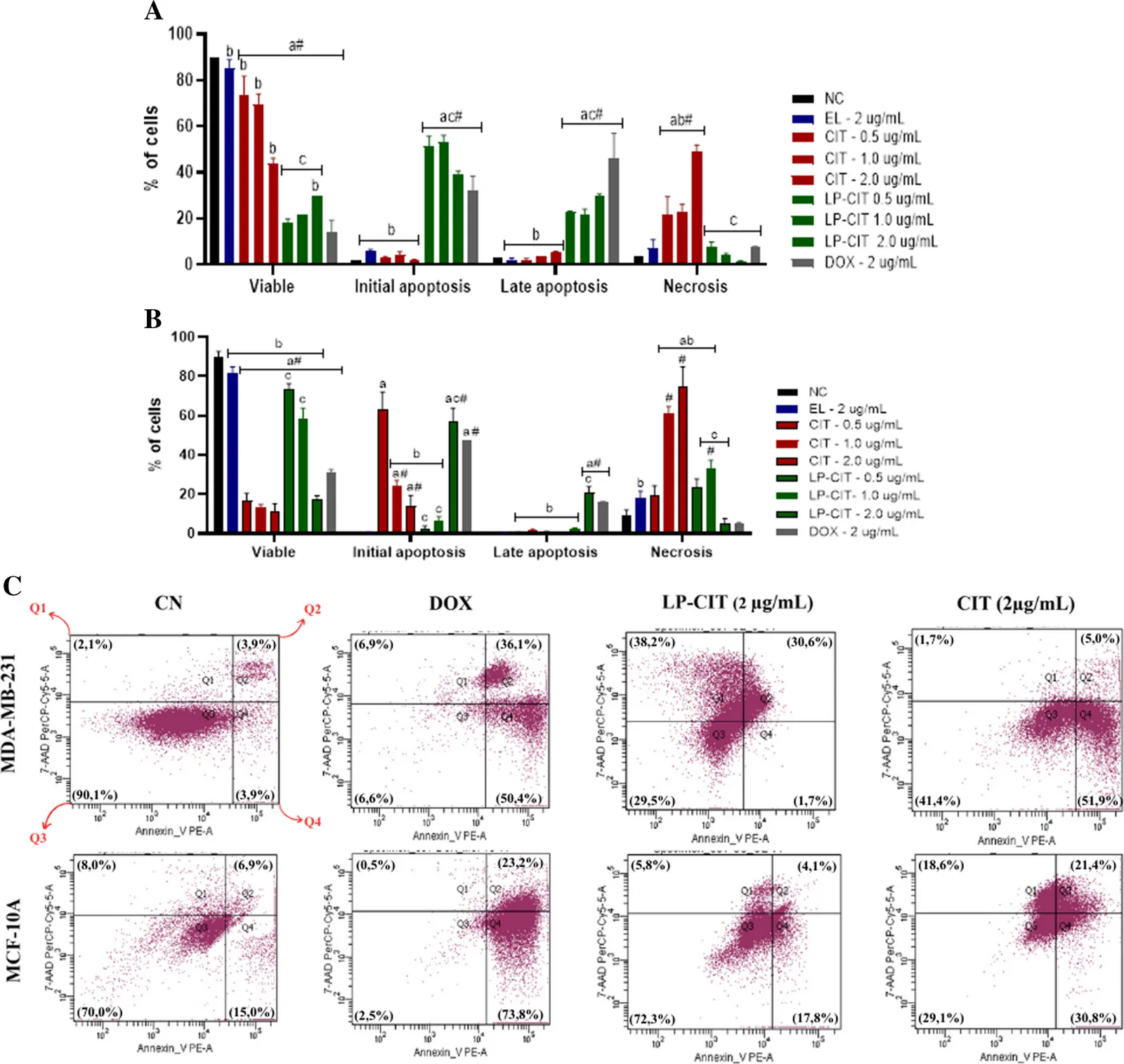
Cytotoxicity and type of cell death by flow cytometry in MDA-MB-231 (**A**), MCF-10A (**B**) and their plots (**C**)

Flow cytometry was conducted in the MDA-MB-231 and MCF-10A cell lines. CIT and LP-CIT induced apoptosis/necrosis at all three concentrations, reducing cell viability for MDA-MB-231 cells (CIT 73.9, 69.5, and 43.8%; LP-CIT 18.2, 21.4, and 29.5%) and MCF-10A (CIT 16.7, 13.4, and 10.9%; LP-CIT 73.6, 58.1, and 17.3%) when compared to NC (90.1%). LP-CIT showed superior results in inducing cell death in comparison to CIT in MDA-MB-231 cells, exhibiting increased apoptosis and reduced necrosis, which indicates better efficiency, which can be validated in forthcoming in vivo experiments (Fig. 3A).

One-way ANOVA followed by Tukey’s post hoc test was performed, and values with *p* < 0.05 were considered statistically significant. Multiple comparisons were conducted among the groups at the same concentration: CN (a), DOX (b), CIT (c), and LV (#). CN, untreated cells. DOX, cells treated with doxorubicin (2 µg/mL). In plots (4C), Q1 represents cells in necrosis, Q2 late apoptosis, Q3 viable cells, and Q4 early apoptosis; in addition, the percentages of cells in each of these conditions are contained within each quadrant.

Regarding initial apoptosis, LP-CIT and DOX significantly increased cell death compared to CN and CIT in MDA-MB-231 cells. In MCF-10A cells, only the highest concentration of LP-CIT (2 µg/mL) showed increased initial apoptosis compared to CIT. Similar results were found for late apoptosis, with LP-CIT (2 µg/mL) showing higher rates of late apoptosis compared to CIT (Fig. 3B). Regarding DOX, all treatment groups (EL, CIT, and LP-CIT) showed higher cell viability, except for the 0.5 and 1 µg/mL concentrations of LP-CIT that did not show a statistically significant difference from DOX (Fig. 3A).

In MCF-10A cells, only LP-CIT concentrations of 0.5 and 1 µg/mL showed higher cell viability than DOX, while the other concentrations of CIT and LP-CIT (2 µg/mL) presented higher cell death (Fig. 3B). The results of late apoptosis in MDA-MB-231 cells were comparable to those observed in early apoptosis, while in MCF-10A cells, only 2 µg/mL of LP-CIT presented higher rates of late apoptosis than CIT. All CIT concentrations showed significantly increased necrosis compared to the other groups in MDA-MB-231 cells. In MCF-10A cells, LP-CIT (1 and 2 µg/mL) showed less necrosis than CIT at the same concentrations. Finally, EL (2 µg/mL) did not show significant differences compared to CN in both cell lines (Fig. 3A).

**Fig. 4:**
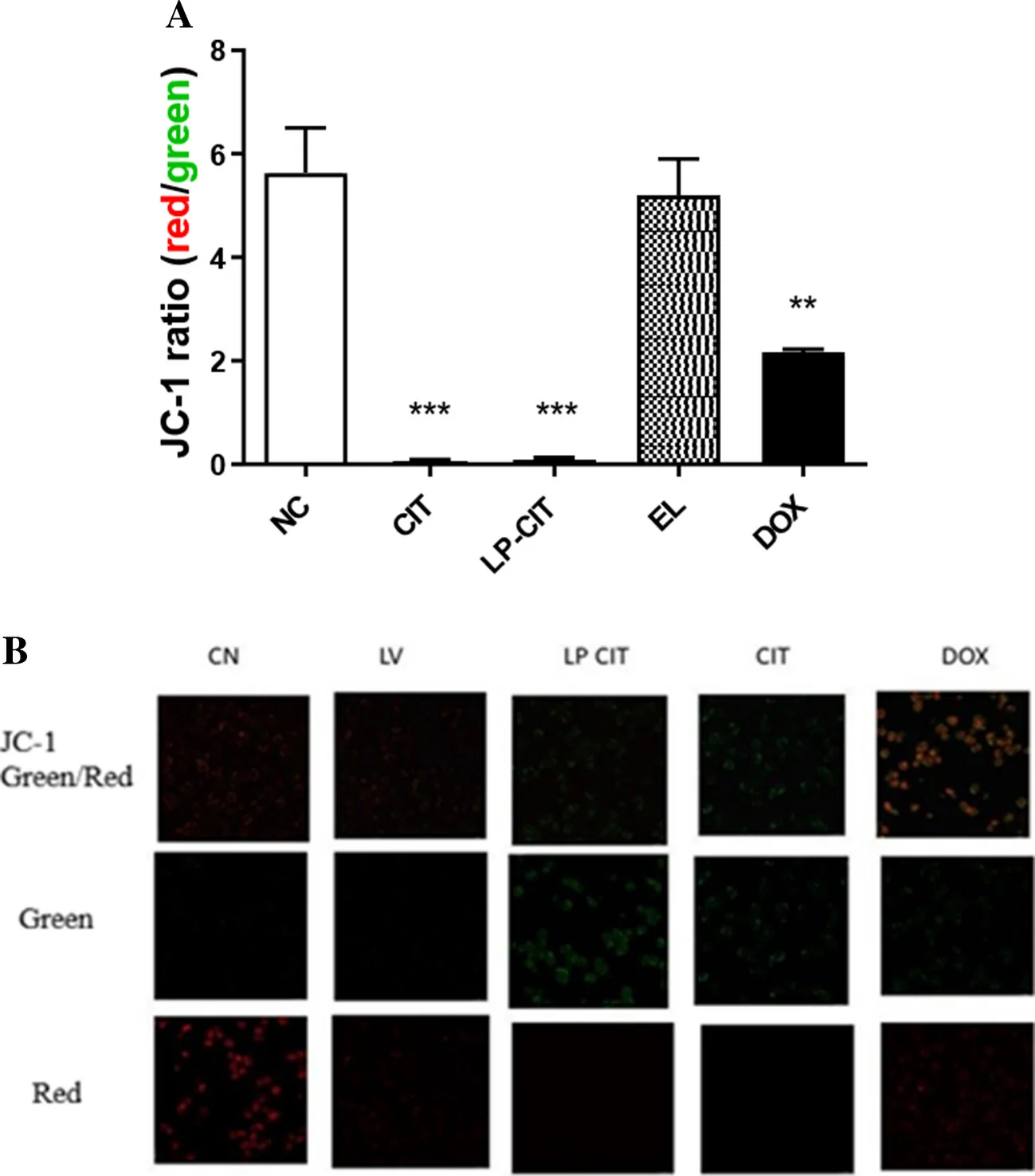
Cell death by mitochondrial membrane potential (JC-1) in MDA-MB-231 cells

The mitochondrial depolarization of MDA-MB-231 cells was assessed using confocal microscopy, indicating apoptosis. Our investigation revealed that the empty liposome did not significantly decrease cell viability of breast tumor cells in comparison to the negative control. Treatment with DOX, free CIT, and LP-CIT markedly reduced cell viability by elevating apoptotic cells observed by the JC-1 marker in its monomeric form, in comparison to the negative control group (Fig. 4).

One-way ANOVA followed by Tukey’s post hoc test was performed, and values with *p* < 0.05 were considered statistically significant. Multiple comparisons were conducted among the groups: CN (**A**) and DOX (**B**). CN, untreated cells. DOX, cells treated with doxorubicin (2 µg/mL). LV, empty liposomes. CIT, free citrinin. LP-CIT, liposomes containing citrinin.

**Fig. 5:**
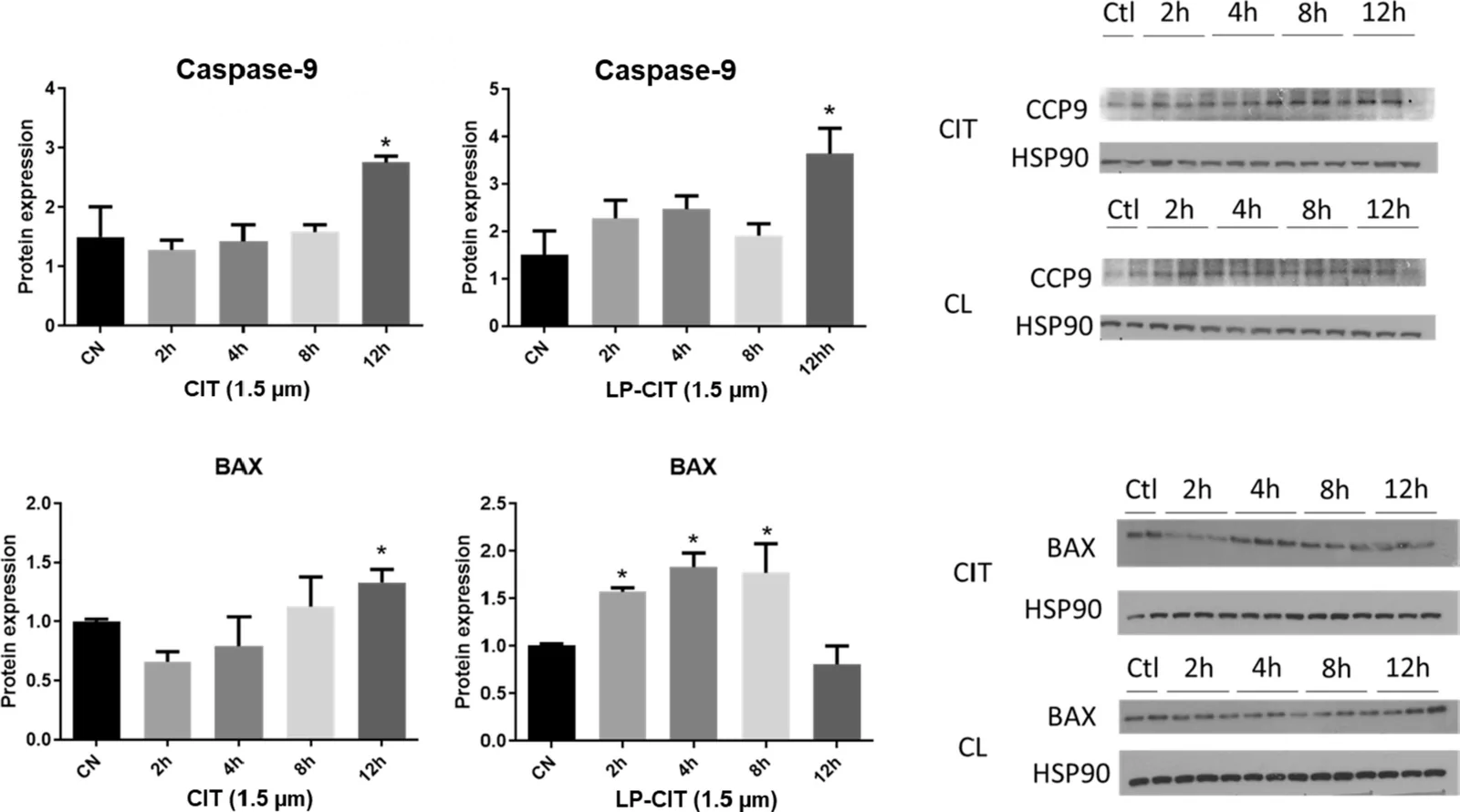
BAX and caspase-9 protein expression in MDA-MB-231 after exposure to CIT and LP-CIT for 2, 4, 8, and 12 h

Western blot analysis showed that caspase 9 was upregulated by CIT and LP-CIT treatment only at 12 h (Fig. 5). In relation to the BAX protein, LP-CIT treatment significantly increased expression at all times analyzed in the study (2–12 h) while CIT increased only at 12 h (Fig. 5).

Protein expression of caspase-9 and BAX in MDA-MB-231 cells after exposure to free citrinin (CIT) or liposomal citrinin (LP-CIT) (1.5 µM) for 2, 4, 8, and 12 h. Protein levels were quantified by densitometric analysis and normalized to HSP90. Data are expressed as mean ± SD of independent experiments. One-way ANOVA followed by Tukey’s post hoc test was performed. *p < 0.05 compared with the control group.

### CIT and LP-CIT genotoxicity

All concentrations of CIT, LP-CIT, and DOX induced genotoxicity in MDA-MB-231 and MCF-10A cells by increasing damage index and frequency when compared to NC. When compared to DOX, in MDA-MB-231 cells, LP-CIT (2 µg/mL) was more genotoxic as assessed by damage index, and CIT (2 µg/mL) and LP-CIT (1 and 2 µg/mL) were more genotoxic as assessed by damage frequency. In MCF-10A cells, CIT (2 µg/mL) and LP-CIT (2 µg/mL) were more genotoxic than DOX in the damage frequency assessment. In addition, the concentrations of 1 and 2 µg/mL of LP-CIT were significantly more genotoxic than the concentrations of CIT with respect to the damage index in MDA-MB-231 cells. The empty liposome showed no genotoxicity in relation to NC (Table 4).

**Table 4:** Genotoxic effects (damage index and frequency of damage) of NC, EL, CIT (0.5, 1.0, and 2.0 µg/mL), LP-CIT (0.5, 1.0, and 2.0 µg/mL), and DOX in breast tumor cells (MDA-MB-231) and and non-tumoral cells (MCF-10A) by alkaline version comet bioassay

		MDA-MB-231		MCF-10A	
	Concentrations	DI	FD	DI	FD
**NC**	-	20.21 ± 3.89	23.50 ± 4.26	15.3 ± 2.3	20.1 ± 3.9
**EL**	2 µg/mL	24.25 ± 7.37	29.25 ± 2.63	18.9 ± 4.7	24.6 ± 2.7
	0.5 µg/mL	128.50 ± 8.12^ab#^	79.50 ± 4.77^ab#^	69.8 ± 7.3^ab#^	80.3 ± 3.6^ab#^
**CIT**	1 µg/mL	148.0 ± 7.89^ab#^	88.00 ± 3.78^ab#^	98.6 ± 8.9^ab#^	89.4 ± 4.2^ab#^
	2 µg/mL	182.40 ± 12.12^ab#^	92.50 ± 5.02ª^#^	132.8 ± 10.2^ab#^	90.8 ± 4.3^a#^
**LP-CIT**	0.5 µg/mL	146.25 ± 15.47^ab#^	80.75 ± 0.95^ab#^	69.7 ± 7.1^ab#^	76.8 ± 3.5^ab#^
	1 µg/mL	207.25 ± 13.50^abc#^	93.50 ± 2.88ª^#^	110.3 ± 6.0^ab#^	85.4 ± 2,0.1^ab#^
	2 µg/mL	290.5 ± 15.78^ac#^	98.50 ± 0.57ª^#^	144.3 ± 9.1^ab#^	91.3 ± 2.4^a#^
**DOX**	2 µg/mL	298.50 ± 13.56^a#^	97.50 ± 1.23^a#^	289.7 ± 10.4^a#^	98.3 ± 2.9^a#^

Values were expressed by mean ± standard deviation using one-way ANOVA and Tukey, with *p*-values < 0.05 statistically significant.

^a^Different when compared to NC.

^b^Different when compared to DOX.

^c^LP-CIT different when compared to CIT at the same concentration.

^#^Different from EL.

*DI* damage index (0–400). *FD* damage frequency (%). *NC* untreated cells. *DOX* cells treated with doxorubicin (2 µg/mL). *CIT* free citrinin. *LP-CIT* liposome containing citrinin. *EL* empty liposome.

## Discussion

Although citrinin is a nephrotoxic and hepatotoxic mycotoxin, its potent cytotoxic activity against cancer cells justifies its investigation as a bioactive lead compound rather than as a systemic drug. Considering that classical chemotherapeutics also exhibit high toxicity, liposomal encapsulation was explored as a strategy to modulate citrinin biological effects, potentially reducing systemic side effects and enhancing tumor accumulation via the enhanced permeability and retention (EPR) effect (Menezes et al. 2023; Moura et al. 2024). Physicochemical characterization confirmed successful nanoscale formulation, supporting subsequent biological evaluations. Particle size distribution was assessed by dynamic light scattering (DLS) (Malvern 2014).

In our study, liposomes were classified as small unilamellar vesicles, exhibiting low polydispersity index and particle size below 150 nm, supporting safe applicability for in vitro assays. The hydrodynamic diameter measured by DLS was 143.3 nm, remaining within the optimal size range for tumor targeting (Qin et al. 2018). The zeta potential of LP-CIT was + 28.5 mV ± 5.79, which is close to the commonly cited ± 30 mV threshold for electrostatic stabilization (Couvreur et al. 2002). Although slightly below this conventional value, no visible aggregation or physicochemical instability was observed during the 21-day evaluation period, indicating adequate short-term stability. Zeta potential plays an important role in nanoparticle suspension stability and cellular interactions (Lin et al. 2021; Smith et al. 2017; Zhu et al. 2020; Zhao et al. 2018), while cellular uptake is also influenced by particle size (Bonferoni et al. 2017). Under physiological conditions, surface charge may be modified by protein corona formation, potentially affecting in vivo stability and biodistribution. LP-CIT presented a PDI of 0.3, indicating acceptable size uniformity (Zhang and Kosaraju 2007). Slight increases in particle size over 21 days (approximately 5–12 nm) were observed but did not indicate progressive aggregation, and PDI values remained below 0.4 without continuous increase. Together, these findings indicate adequate short-term physicochemical stability without compromising formulation reproducibility under the evaluated storage conditions.

pH-sensitive liposomes remain stable at physiological pH (7.4) and may release their cargo under acidic tumor microenvironment conditions due to lipid bilayer destabilization (Riaz et al. 2018). Liposomal drug delivery systems are widely used to improve pharmacokinetic properties and modulate drug distribution and toxicity (Eloy et al. 2014; Guo and Huang 2014; Riaz et al. 2018; Sharma et al. 2015; Bregoli et al. 2016). In the present study, CIT and LP-CIT were evaluated for cytotoxicity in breast tumor cell lines, with LP-CIT demonstrating increased cytotoxicity across all tested lines. Previous studies have reported cytotoxic effects of CIT in multiple cancer models (Yao et al. 2011; Oliveira et al. 2020b; Yazdani et al. 2012). The cytotoxicity observed for empty liposomes (EL) required careful interpretation, as cationic lipids may interfere with MTT assays due to interactions with lipid droplets (Angius and Floris 2015). EL-associated cytotoxicity was mainly detected at higher concentrations and was not consistently confirmed by trypan blue or flow cytometry, suggesting potential assay interference rather than true toxicity. The positive surface charge conferred by octadecylamine may also promote electrostatic interactions with negatively charged cell membranes. Importantly, empty liposomes did not induce genotoxic effects, indicating that DNA damage observed after LP-CIT exposure is predominantly attributable to citrinin (Andrade et al. 2022). Therefore, although MTT assays were used for initial LC₅₀ estimation, trypan blue and flow cytometry were considered more reliable endpoints. These differences likely reflect the distinct principles of each assay, as MTT evaluates metabolic activity whereas trypan blue and flow cytometry assess membrane integrity and apoptotic processes more directly.

Several molecular markers have been used to evaluate cytotoxicity and apoptosis mechanisms, including caspase activation and PARP cleavage (Casili et al. 2020; Tixeira et al. 2020; Wang et al. 2019). Previous studies demonstrated that CIT induces apoptosis through mitochondrial dysfunction, ROS generation, and activation of intrinsic apoptotic pathways (Chen and Chan 2009; Gayathri et al. 2015). In the present study, CIT and LP-CIT increased expression of BAX and caspase-9 in MDA-MB-231 cells, supporting activation of the intrinsic mitochondrial pathway (Peña‐Blanco and García 2018; Aliabadi et al. 2021; Bratton and Salvesen 2010; Avrutsky and Troy 2021; Kaplowitz 2000). These findings are consistent with previous reports describing cytochrome C release and caspase activation following CIT exposure (Yu et al. 2006; Gayathri et al. 2015). Although downstream executors such as caspase-3 or PARP cleavage were not evaluated, the combined increase in BAX expression, caspase-9 activation, mitochondrial depolarization, and Annexin V positivity strongly supports activation of the intrinsic apoptotic pathway.

The results of flow cytometry pointed out CIT and LP-CIT’s ability to reduce cell viability in the mammary tumor cell line MDA-MB-231 and in the non-tumor cell line MCF-10A. LP-CIT showed better results regarding cell death compared to free CIT in MDA-MB-231 cells, with higher percentages of late apoptosis and lower levels of necrosis, indicating greater efficacy for potential antitumor activity (Fig. 3). Flow cytometry results demonstrated that LP-CIT was more cytotoxic to MDA-MB-231 tumor cells than CIT. Interestingly, in non-tumor MCF-10A cells, LP-CIT was less cytotoxic than CIT at concentrations of 0.5 and 1 µg/mL, demonstrating higher affinity for tumor cells rather than non-tumor cells. Overall, the liposomal formulation primarily increased the biological potency of citrinin rather than clearly improving its therapeutic window, as cytotoxic and genotoxic effects were also observed in non-tumorigenic cells. This limited improvement in tumor selectivity may be attributed to the non-specific cellular uptake of cationic liposomes, whose positive surface charge promotes electrostatic interactions with negatively charged membranes in both tumor and non-tumor cells. As no active targeting strategy was employed, nanoencapsulation likely enhanced overall intracellular delivery rather than tumor-specific accumulation. Therefore, the main contribution of nanoencapsulation in the present study was enhancement of cytotoxic potency rather than a substantial widening of the therapeutic window. These findings reinforce that additional targeting strategies or surface functionalization may be necessary to improve tumor selectivity in future studies.

Apoptosis induction is frequently associated with mitochondrial membrane depolarization, which can be assessed using JC-1 staining (Zollet et al. 2021; Carneiro and El-Deiry 2020; Kalkavan and Green 2018). In this study, empty liposomes did not significantly alter mitochondrial membrane potential in MDA-MB-231 cells, whereas CIT, LP-CIT, and DOX induced significant mitochondrial depolarization consistent with apoptotic activation. Similar mitochondrial effects of CIT have been described in HCT116, HEK293, and embryonic mouse cells, where apoptosis was associated with ROS generation and caspase activation (Salah et al. 2019, 2017; Chang et al. 2009; Chan and Shiao 2007).

The cytotoxicity observed for CIT and LP-CIT was associated with genotoxic effects demonstrated by comet assay, revealing DNA fragmentation in both tumor (MDA-MB-231) and non-tumor (MCF-10A) cells. Previous studies similarly reported CIT-induced DNA damage, chromosomal abnormalities, and genotoxic responses in different cellular models (Bouslimi et al. 2008; Anninou et al. 2014; Dönmez-Altuntas et al. 2007; Knasmuller et al. 2004). Although mutagenicity was not directly assessed, the observed DNA damage raises safety concerns and may negatively impact the therapeutic index. LP-CIT induced more severe DNA damage compared with free CIT, likely reflecting enhanced intracellular delivery associated with nanoencapsulation (Moura et al. 2024). Overall, nanoencapsulation potentiated cytotoxic and genotoxic effects but showed limited tumor selectivity.

The primary genotoxic mechanism of CIT is associated with ROS generation and oxidative stress. Previous studies demonstrated modulation of antioxidant enzymes, increased lipid peroxidation, and oxidative damage across different cell models (Babu et al. 2017; Salah et al. 2017; Oliveira et al. 2017). DNA fragmentation observed in the present study is consistent with ROS-mediated apoptosis and late apoptotic events (Wang et al. 2016; Jamin et al. 2003).

Certain endpoints exhibited relatively broad standard deviations or confidence intervals, reflecting biological variability inherent to cellular systems as well as technical factors associated with experimental procedures. Higher SDs observed in zeta potential measurements may derive from differences in particle preparation and measurement sensitivity. Moderately wide confidence intervals in LC₅₀ values, particularly in MDA-MB-231 cells, likely reflect heterogeneous cellular responses. The comet assay showed greater variability at higher concentrations, which may be associated with stochastic DNA damage responses under elevated stress conditions. Despite this variability, consistent trends across independent biological replicates support the reliability and robustness of the observed effects.

The results of the present study indicated that citrinin and its liposomal formulation can induce cell death predominantly through apoptosis. Liposomal encapsulation enhanced the biological effects of citrinin in tumor cells, resulting in increased cytotoxic and genotoxic activity. However, the findings also reveal important safety limitations, as relevant cytotoxic and genotoxic effects were observed in non-tumor cells, indicating low tumor selectivity. These results highlight that, despite the increased biological activity promoted by the nanoformulation, LP-CIT still requires further experimental testing before it can be considered a candidate antineoplastic drug. As a result, the current study should be interpreted primarily as a genotoxic evaluation and a proof-of-concept formulation study, with the goal of understanding how liposomal encapsulation affects the biological profile of citrinin in vitro, rather than as a recommendation for near-term therapeutic use. Future studies should include in vivo assessment of biodistribution, systemic toxicity, and therapeutic index under physiological conditions, as well as investigation of targeting strategies or surface functionalization to improve tumor selectivity and minimize off-target effects. Further studies should also include additional physicochemical and pharmacokinetic characterization, such as drug release profiling and serum stability evaluation, to better support the translational development of LP-CIT.

## Conclusion

Citrinin demonstrated cytotoxic and genotoxic activity against the evaluated mammary tumor and non-tumor cell lines. Furthermore, the liposomal formulation (LP-CIT) showed better cytotoxic and genotoxic effects at the same concentrations compared to free citrinin. These results indicate that citrinin, with an emphasis on LP-CIT at low concentrations (0.5–2.0 µg/mL), produces more pronounced toxic effects against mammary cell lines, which should be evaluated in vivo to determine the already known systemic toxicity of this marine metabolite.

## Supplementary Information

Below is the link to the electronic supplementary material.

ESM 1 (JPG 69.6 KB)

ESM 2 (JPG 39.9 KB)

ESM 3 (JPG 50.7 KB)

ESM 4 (JPG 6.13 KB)

ESM 5 (JPG 6.13 KB)

ESM 6 (JPG 27.7 KB)

ESM 7 (JPG 8.98 KB)

ESM 8 (JPG 8.98 KB)

ESM 9 (JPG 14.4 KB)

ESM 10 (JPG 12.7 KB)

ESM 11 (JPG 12.7 KB)

(PNG 1.66 MB)

ESM 12 (TIF 2.16 MB)

## Data Availability

The data underlying this article will be shared on reasonable request to the corresponding author.
